# Acute Effects of Exercise on Metabolic, Inflammatory, and Immune Markers in Adolescent Girls with Normal Weight or Overweight/Obesity

**DOI:** 10.3390/sports14010024

**Published:** 2026-01-05

**Authors:** Wissal Abassi, Nejmeddine Ouerghi, Moncef Feki, Santo Marsigliante, Anissa Bouassida, Beat Knechtle, Jolita Vveinhardt, Antonella Muscella

**Affiliations:** 1 Research Unit “Sport Sciences, Health and Movement” (UR22JS01) High Institute of Sport and Physical Education of Kef, University of Jendouba, Kef 7100, Tunisia; wissalabassi93@gmail.com (W.A.); najm_ouerghi@hotmail.com (N.O.); bouassida_anissa@yahoo.fr (A.B.); 2 Faculty of Medicine of Tunis, Rabta Hospital, University of Tunis El Manar, LR99ES11, Tunis 1007, Tunisia; monssef.feki@gmail.com; 3 Department of Biological and Environmental Science and Technologies (DiSTeBA), University of Salento, 73100 Lecce, Italy; santo.marsigliante@unisalento.it; 4 Institute of Primary Care, University Hospital of Zurich, 8091 Zurich, Switzerland; beat.knechtle@hispeed.ch; 5 Medbase St. Gallen Am Vadianplatz, 9000 St. Gallen, Switzerland; 6 Klaipėdos Valstybinė Kolegija/Higher Education Institution, 91274 Klaipeda, Lithuania

**Keywords:** adolescent girls, obesity, Spartacus exercise, glucose, total cholesterol, C-reactive protein, leukocytes, acute exercise, metabolic response, immune response

## Abstract

**Background:** Obesity alters metabolic, inflammatory, and immune responses, and acute exercise may affect these parameters differently according to body composition. This study investigated the acute effects of Spartacus exercise on metabolic, inflammatory, and immune markers in adolescent girls with overweight/obesity and normal weight. **Methods:** In this non-randomized clinical study, sixteen girls with overweight/obesity (BMI: 31.17 ± 3.85 kg/m^2^) and fourteen normal-weight girls (BMI: 21.93 ± 0.99 kg/m^2^) performed an intermittent running test (15 s effort, 15 s passive recovery), starting at 7 km·h^−1^ with 1 km·h^−1^ increments every 3 min until exhaustion. Blood samples were collected at rest (T0), immediately post-exercise (T1), and 30 min post-exercise (T2). CRP and ESR were assessed at baseline to characterize participants’ inflammatory status, while glucose and leukocyte subpopulations were evaluated to investigate acute exercise responses. **Results:** Fasting glucose, lipid profile (TC, TG, HDL-C, LDL-C), inflammatory markers (CRP, ESR), and leukocyte subpopulations were assessed. Significant group effects were observed for all metabolic and inflammatory markers, reflecting higher baseline values in participants with overweight/obesity compared with normal-weight participants (*p* < 0.05). Significant effects of time were found for glucose and leukocytes (*p* < 0.001), indicating acute exercise-induced changes, along with significant time × group interactions. Participants with overweight/obesity showed greater and more prolonged increases in glucose, total leukocytes, and neutrophils, whereas normal-weight girls returned to baseline within 30 min. **Conclusions:** Acute high-intensity intermittent exercise induces transient metabolic and immune responses in adolescents, with amplified and prolonged effects in those with obesity. These findings highlight the importance of considering body composition when prescribing exercise programs.

## 1. Introduction

The prevalence of childhood and adolescent obesity has reached alarming levels, representing a major global public health concern in the 21st century. A 2025 UNICEF–WHO–World Bank joint analysis estimated that 1 in 5 children and adolescents aged 5–19 years (about 391 million) are overweight, and for the first time in history, obesity now surpasses underweight among school-aged children and adolescents in most regions, with roughly 1 in 10 (about 188 million) classified as obese [1]. This alarming trend underscores the rapidly escalating burden of excess weight in young populations worldwide and the urgent need for coordinated public health action.

Projections are particularly concerning, suggesting that by 2035 nearly one-quarter of children and adolescents worldwide (~25%) will be overweight or obese, with the number of youths with obesity expected t exceed 360 million by 2050 [2].

The dynamic nature of the obesity epidemic has been further amplified in the wake of the COVID-19 pandemic. Evidence shows that lockdowns and pandemic-related restrictions were associated with marked reductions in physical activity, increases in sedentary behavior, and deteriorations in dietary habits among children and adolescents, contributing to increases in BMI and the prevalence of overweight and obesity in this population [3,4,5].

Excess adiposity during adolescence is associated with a range of metabolic and physiological disturbances, including insulin resistance, dyslipidemia, and impaired glucose homeostasis [6,7]. These alterations contribute to endothelial impairment, pro-inflammatory immune activation, and the early onset of cardiometabolic diseases [8,9,10]. Moreover, chronic low-grade systemic inflammation, adipose tissue dysfunction, and altered adipokine secretion have been observed in adolescents with obesity, further amplifying metabolic and vascular stress and highlighting the heightened physiological vulnerability in this population [11,12,13].

Regular physical activity is recognized as a cornerstone intervention for improving insulin sensitivity, regulating hormonal balance, reducing systemic inflammation, enhancing lipid metabolism, and promoting overall cardiovascular health in youth with overweight and obesity [14,15,16]. However, evidence regarding the acute physiological responses to a single maximal incremental exercise bout remains limited, as most studies focus on chronic interventions [17]. Acute exercise can transiently influence glucose homeostasis, lipid metabolism, and leukocyte distribution, with responses potentially differing between normal-weight adolescents and those with overweight/obesity, reflecting baseline metabolic and inflammatory alterations associated with excess adiposity [18,19].

Understanding these immediate responses is particularly relevant in this population because adolescent girls with overweight/obesity often present baseline alterations in glucose metabolism, inflammatory status, and immune function, which may amplify or prolong responses to high-intensity exercise. Characterizing these acute changes can provide critical insight for tailoring exercise prescriptions, optimizing safety, and maximizing metabolic and immune benefits in youth at risk of cardiometabolic complications.

Evidence from pilot studies of high-intensity interval training (HIIT) in adolescents with overweight/obesity supports this notion: acute HIIT has been shown to induce immediate post-exercise elevations in IL-6 and cortisol [20], and interval training can significantly modulate insulin resistance and glucose regulation in female adolescents with obesity [21]. These findings highlight the rapid metabolic, inflammatory, and immune responses triggered by high-intensity exercise in youth with varying BMI. Beyond these acute effects, longer-term HIIT interventions have been shown to produce substantial improvements in cardiometabolic health in adolescents with overweight or obesity. Specifically, HIIT has been reported to enhance cardiorespiratory fitness (VO_2_max and VO_2_peak), reduce body fat, BMI, and visceral adipose tissue, improve lipid profiles (LDL, total cholesterol, triglycerides, HDL), and decrease HOMA-IR, while also positively affecting adipokine profiles [22,23,24,25]. Despite these well-documented benefits, concerns have been raised regarding the tolerability and acceptability of high-intensity exercise in adolescents with overweight/obesity, particularly due to elevated perceived exertion and potentially lower enjoyment compared with moderate-intensity protocols. For example, a 12-week HIIT program in overweight/obese female adolescents produced improvements in fitness and body composition, but participants reported higher perceived exertion and lower enjoyment scores than those in a moderate-intensity interval training group, indicating potential psychological barriers to long-term adherence without appropriate support and motivation strategies [26]. In addition, long-term attendance and retention can be problematic; in some HIIT interventions, attendance rates decreased substantially over time, and high dropout has been observed in unsupervised or minimally supported programs, highlighting adherence challenges in this population [27].

However, other research suggests that when HIIT is appropriately supervised, individualized, and embedded within a supportive framework, adherence can be relatively high and dropout rates may be lower than in non-exercise control conditions, supporting its feasibility when barriers are proactively managed through supervision and motivational support [28]. Consequently, careful supervision, gradual progression, individualized exercise prescription, and attention to psychological factors such as enjoyment, perceived exertion, and motivation are recommended to maximize the metabolic and cardiometabolic benefits of HIIT while minimizing potential barriers to participation among adolescents with overweight/obesity.

These findings highlight the critical role of body composition in shaping physiological responses to high-intensity exercise and underscore the need to understand immediate metabolic, inflammatory, and immune reactions in youth with differing BMI.

This study investigates the acute effects of a maximal Spartacus exercise protocol on glucose, lipid profile, and immune responses in adolescent girls with normal weight versus overweight/obesity. Leukocyte subpopulations were measured to capture rapid, exercise-induced immune changes, while inflammatory markers (C-reactive protein [CRP] and erythrocyte sedimentation rate [ESR]) were included to reflect baseline systemic inflammation associated with overweight/obesity, rather than acute responses.

We hypothesize that acute exercise will induce transient increases in glucose and leukocytes in all participants, and that adolescents with overweight/obesity will show greater and more prolonged metabolic and immune responses, reflecting heightened baseline metabolic and inflammatory stress.

These differential responses are expected to underscore the importance of considering body composition when prescribing high-intensity exercise in youth. By addressing these hypotheses, the study provides practical insights for designing safe, effective, and individualized exercise programs aimed at promoting cardiometabolic health and preventing obesity in adolescents.

## 2. Materials and Methods

### 2.1. Study Design

**Figure 1:**
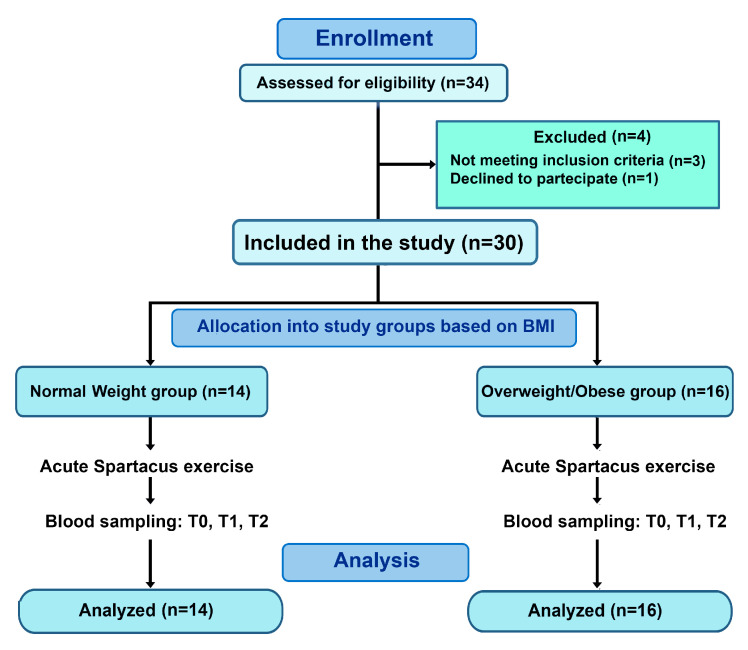
Flow diagram of participant recruitment and allocation. Thirty participants were included and allocated based on BMI into a normal-weight group (BMI: 21.93 ± 0.99 kg/m^2^) and an overweight/obesity group (BMI: 31.17 ± 3.85 kg/m^2^). All participants completed the acute Spartacus exercise protocol and blood sampling at T0, T1, and T2.

The study employed a non-randomized, controlled design. Participants were allocated based on BMI and all completed the same acute exercise protocol. The experiment was conducted over two separate laboratory sessions, with all sessions scheduled at 08:00 a.m. During the first session, anthropometric parameters—including height, body mass, body mass index, body fat percentage, and waist circumference—were assessed in lightly clothed, barefoot participants using standard procedures commonly reported in pediatric exercise and obesity research [22,29]. Participants were also familiarized with the Spartacus exercise protocol. After a 48-h interval, the experimental session was performed. Participants arrived at the laboratory in the morning following an overnight fast of at least 10 h. A baseline venous blood sample (T0) was collected prior to the Spartacus exercise test, with additional samples obtained immediately post-exercise (T1) and 30 min post-exercise (T2) (Figure 1). All procedures were conducted under standardized laboratory conditions to ensure consistency across participants. **ClinicalTrials.gov Identifier:** NCT07103889.

### 2.2. Participant

Thirty-four adolescent girls aged 15–18 years were initially recruited from local secondary schools. Recruitment was conducted via announcements and invitations sent to students and their parents or guardians. All interested participants were screened according to predefined inclusion and exclusion criteria, and eligible adolescents were subsequently assigned to either the normal-weight or overweight/obese group based on BMI.

Four participants were excluded before baseline assessment due to: (i) not meeting the inclusion criteria and/or (ii) declining to participate. The final sample comprised thirty participants.

Inclusion criteria were: (i) female sex, (ii) age between 15 and 18 years, (iii) absence of regular structured training (>2 sessions/week) in the previous six months, and (iv) medical clearance to participate in high-intensity exercise. Exclusion criteria included: (i) history of chronic metabolic, cardiovascular, or inflammatory diseases, (ii) recent infection or use of medications affecting immune or metabolic function within the past four weeks, and (iii) use of hormonal contraception.

Participants were categorized into two groups based on body mass index (BMI): adolescents with overweight/obesity (*n* = 16; BMI: 31.17 ± 3.85 kg/m^2^) and normal-weight adolescents (*n* = 14; BMI: 21.93 ± 0.99 kg/m^2^) (Figure 1). Due to visible differences between groups, full blinding of all procedures was not feasible. To minimize potential bias, laboratory personnel conducting biochemical analyses were blinded to group allocation, whereas researchers performing anthropometric measurements and exercise testing were aware of group classification. In addition, all participants, including those in the control/normal-weight group, underwent the same procedural conditions, including standardized visit timing and blood sampling, to minimize the influence of circadian rhythms. Standardized instructions were provided to all participants to reduce procedural stress. These limitations are acknowledged in the study design

Before recruitment, an a priori sample size calculation was conducted using G*Power software (version 3.1.9.4, University of Kiel, Kiel, Germany) for a repeated-measures ANOVA with within–between interaction. Effect sizes were estimated from previous studies investigating acute exercise-induced metabolic and inflammatory responses in adolescents with overweight/obesity and normal weight [23,30,31]. The calculation was based primarily on the glucose response to acute exercise, assuming a large effect size (f = 0.40), an alpha level of 0.05, and a desired power of 0.80, which indicated a minimum of 26 participants. To account for potential dropouts, 30 participants were recruited. We acknowledge that the study may have limited power to detect changes in inflammatory markers (CRP and ESR), given their slower kinetics and smaller expected effect sizes.

All participants and their parents or legal guardians were fully informed about the study procedures and potential risks, and written informed consent was obtained prior to participation. To further control for potential confounding effects related to circadian rhythms or procedural stress, participants were instructed to maintain usual daily routines and were scheduled at similar times of day for testing. Participants were also instructed to avoid vigorous physical activity, maintain their usual diet, and ensure adequate sleep for at least 24 h before each laboratory session.

### 2.3. Spartacus Exercise Protocol

**Figure 2:**
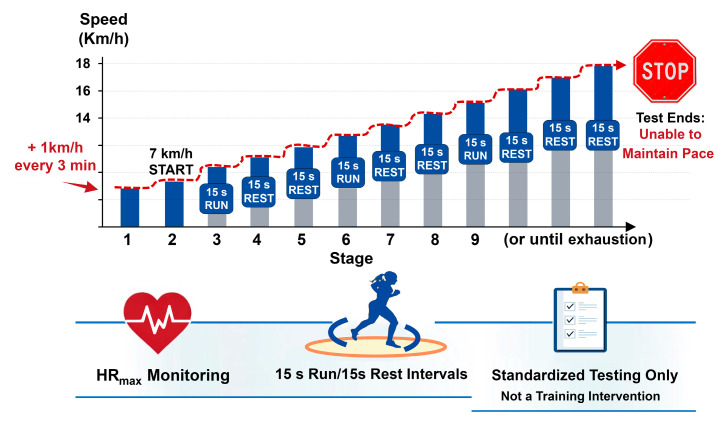
**Spartacus exercise protocol.** The test was performed on a 750 m rectangular track (75 × 10 m) and consisted of incremental running stages starting at 7 km·h^−1^. Running speed increased by 1 km·h^−1^ every 3 min up to 18 km·h^−1^. Within each stage, participants alternated 15 s of running at the prescribed speed with 15 s of passive recovery, paced by an audio signal. The test was terminated when participants were no longer able to maintain the required running speed.

The Spartacus test was conducted on a 750 m rectangular track (75 × 10 m), marked with fixed cones positioned at regular intervals corresponding to target running speeds ranging from 7 to 18 km·h^−1^. The protocol started at 7 km·h^−1^, with increments of 1 km·h^−1^ every 3 min. Within each stage, participants alternated 15 s of running to reach the designated marker with 15 s of passive recovery. Running pace was externally regulated by a standardized audible signal, and participants were instructed to reach the corresponding cone exactly at the sound of the signal, ensuring synchronization between distance covered and time elapsed. To reduce pacing variability and potential errors related to uneven running speed within each section, all trials were continuously supervised by experienced investigators, who provided immediate verbal corrections if participants arrived early or late at the designated marker. Failure to maintain synchronization with the auditory signal despite feedback was considered an inability to sustain the required pace and resulted in test termination. The test was stopped when participants could no longer maintain the prescribed running speed [32,33] (Figure 2). It was performed to measure speed associated with VO_2peak_ (maximal aerobic speed [MAS]) and to provide a standardized acute exercise stimulus. This protocol has been validated for assessing aerobic capacity in obese adolescents [34], and was used solely as a testing procedure, not as a training intervention [35]. Maximal heart rate (HRmax) was continuously monitored throughout the test using a heart rate monitor (S810; Polar, Kempele, Finland). Although participants’ subjective perception of effort was not formally recorded in this study, the use of the Borg Rating of Perceived Exertion (RPE) scale could provide valuable information regarding individual exercise tolerance and is recommended for future studies.

### 2.4. Blood Sampling and Biochemical Analysis

Venous blood samples were collected in the morning after a 12-h overnight fast at three time points: at rest before exercise (T0), immediately after completion of the Spartacus test (T1), and 30 min post-exercise (T2). Sampling was performed from an antecubital vein using standard aseptic procedures, with participants in a seated position. Blood was collected into EDTA tubes for hematological analysis, plain tubes for serum separation, and sodium citrate tubes (blue top) for erythrocyte sedimentation rate (ESR) determination. Samples for serum analysis were allowed to clot at room temperature and subsequently centrifuged at 3000 rpm for 10 min. The resulting serum was aliquoted and stored at −80 °C until analysis.

Biochemical measurements included fasting glucose (GL), C-reactive protein (CRP), total cholesterol (TC), triglycerides (TG), and high-density lipoprotein cholesterol (HDL-C), which were determined using an automated immunoassay system (AU480 Chemistry Analyzer; Beckman Coulter, Brea, CA, USA). Low-density lipoprotein cholesterol (LDL-C) was calculated using the Friedewald formula [36]. ESR was measured using the Westergren method. Leukocyte (LE) subpopulations, including neutrophils (NEU), lymphocytes (LYM), monocytes (MONO), eosinophils (EO), and basophils (BASO), were quantified using an automated hematology analyzer (XN-450; Sysmex, Norderstedt, Germany). CRP and ESR were included to characterize baseline or subacute systemic inflammation, as their kinetics are too slow to detect acute exercise-induced changes within the short post-exercise window. Acute immune responses were assessed via leukocyte subpopulations, which respond rapidly to short-term exercise

To ensure reliability, all assays were performed in duplicate, and intra-assay and inter-assay coefficients of variation were maintained below 5% for all biochemical measurements. Quality control procedures included calibration with standard reference materials and periodic verification of analyzer performance, in accordance with the manufacturer’s guidelines.

### 2.5. Statistical Analysis

Prior to participant recruitment, a sample size calculation was performed using G*Power software (version 3.1.9.4; University of Kiel, Kiel, Germany) based on a repeated-measures ANOVA design with a within-between interaction. The calculation was informed primarily by the expected glucose response to acute exercise, with effect sizes drawn from previous studies with similar participants [21,30,31]. The analysis indicated that a minimum of 16 participants per group (effect size f = 0.40, α = 0.05, power = 0.80) was required to detect significant differences for this primary outcome. We acknowledge that the assumed large effect size may not apply uniformly across all measured variables, particularly inflammatory markers such as CRP and ESR, which are expected to show smaller changes due to their slower kinetics. All statistical analyses were conducted using SPSS software (version 27.0; IBM Corp., Armonk, NY, USA). Although CRP and ESR were included in the repeated-measures ANOVA, these markers were not expected to exhibit acute changes due to their slow kinetics. Their analysis primarily aimed to compare baseline or subacute inflammatory status between normal-weight and overweight/obese participants. Acute immune responses were assessed via leukocyte subpopulations, which respond rapidly to short-term exercise.

Data are presented as mean ± standard deviation (SD), and normality of distributions was assessed using the Shapiro–Wilk test. The assumption of sphericity for repeated-measures ANOVA was tested using Mauchly’s test, and when violations were detected, the Greenhouse–Geisser correction was applied to adjust the degrees of freedom. A two-way repeated-measures ANOVA was applied to examine the main effects of time (T0, T1, T2), group (normal-weight vs. overweight/obese), and their interaction (time × group). When a significant time × group interaction was observed, post hoc analyses were performed only for those variables. Paired samples t-tests were used for within-group comparisons, and independent samples t-tests were used for between-group comparisons at each time point. Given the exploratory nature of some secondary outcomes and the limited number of comparisons per variable, this approach was considered appropriate, although it may increase the risk of Type I error. No formal correction for multiple comparisons was applied, given the exploratory nature of some outcomes. Therefore, results—particularly for secondary outcomes such as inflammatory markers (CRP, ESR) and leukocyte subpopulations—were interpreted with caution to minimize the risk of Type I error. Statistical significance was set at *p* < 0.05. Effect sizes for pairwise comparisons were calculated using Cohen’s d and interpreted as small (0.00–0.49), moderate (0.50–0.79), or large (≥0.80) effects. Additionally, partial eta squared (η^2^p) was calculated for ANOVA to quantify effect magnitude, with thresholds of 0.01 = small, 0.06 = medium, and 0.14 = large effect [37].

## 3. Results

The anthropometric characteristics of the participants are summarized in Table 1. No significant differences were observed between groups for age and height (*p* > 0.05). In contrast, the group with overweight/obesity exhibited significantly higher body mass (*p* < 0.001, d = 2.56), BMI (*p* < 0.001, d = 3.30), body fat percentage (*p* < 0.001, d = 3.88), and waist circumference (*p* < 0.001, d = 4.56) compared to normal-weight participants. Additionally, maximal aerobic speed (MAS) (*p* < 0.001, d = 2.59) and maximal heart rate (HRmax) (*p* = 0.002, d = 1.34) were significantly lower in the group with overweight/obesity.

### 3.1. Correlations Between Adiposity Indices and Metabolic and Inflammatory Markers

Correlation analysis showed that both BMI and body fat percentage were significantly associated with several metabolic and inflammatory parameters (Table 2). Moderate-to-strong positive correlations were observed with glucose, LDL cholesterol, CRP, ESR, and leukocyte counts, while body fat percentage was inversely correlated with HDL cholesterol.

### 3.2. Acute Exercise Effects on Metabolism and Immune Responses

Significant main effects of group were observed for glucose (GL, mmol/L), total cholesterol (TC, mmol/L), triglycerides (TG, mmol/L), high-density lipoprotein cholesterol (HDL-C, mmol/L), low-density lipoprotein cholesterol (LDL-C, mmol/L), C-reactive protein (CRP, mg/L), erythrocyte sedimentation rate (ESR, mm/h), total leukocytes (LE, ×10^3^/µL), neutrophils (NE, ×10^3^/µL), lymphocytes (LYM, ×10^3^/µL), monocytes (MONO, ×10^3^/µL), and basophils (BASO, ×10^3^/µL). These significant group effects primarily reflect baseline differences between normal-weight and overweight/obese adolescents due to BMI status, rather than acute exercise-induced changes.

CRP and ESR were included strictly as indicators of baseline systemic inflammation and should not be interpreted as reflecting acute inflammatory responses. Acute exercise responses were instead captured by time-dependent changes in glucose and leukocyte subpopulations.

Significant main effects of time were found for GL, LE, NE, and LYM, whereas no significant time effects were observed for TC, TG, HDL-C, LDL-C, CRP, ESR, eosinophils (EO, ×10^3^/µL), or BASO (all *p* > 0.05) (Table 3).

Significant time × group interactions were detected for GL (*p* = 0.037), LE (*p* = 0.029), NE (*p* = 0.044), and LYM (*p* = 0.039). Post hoc analyses showed that in adolescents with overweight/obesity, GL increased from pre-test (T0) to immediately post-test (T1; *p* < 0.001) and remained elevated at 30 min post-test (T2; *p* = 0.038). Similarly, LE and NE increased from T0 to T1 (*p* < 0.001 for both) and remained above baseline at T2 (LE: *p* = 0.034; NE: *p* = 0.018). LYM increased at T1 (*p* = 0.003) and returned toward baseline at T2 (*p* > 0.05).

In normal-weight adolescents, GL increased at T1 (*p* < 0.001) and returned to baseline at T2 (*p* = 0.137). LE and NE also increased at T1 (*p* < 0.001 for both) but normalized by T2 (*p* > 0.05). LYM increased at T1 (*p* < 0.001) and decreased below baseline at T2 (*p* < 0.001) (Table 3).

Between-group comparisons confirmed that adolescents with overweight/obesity had higher baseline (T0) values for GL, TC, TG, HDL-C, LDL-C, CRP, ESR, LE, NE, LYM, MONO, and BASO compared to normal-weight peers. These group differences primarily reflect baseline BMI-related differences rather than acute exercise-induced changes. Many of these baseline differences persisted at T1 and T2; however, true acute exercise responses were captured by time-dependent changes in glucose and leukocyte subpopulations, while CRP and ESR values reflect baseline systemic inflammation only.

Effect sizes for post hoc comparisons ranged from moderate to large, highlighting the physiological relevance of these acute metabolic and immune responses.

**Figure 3:**
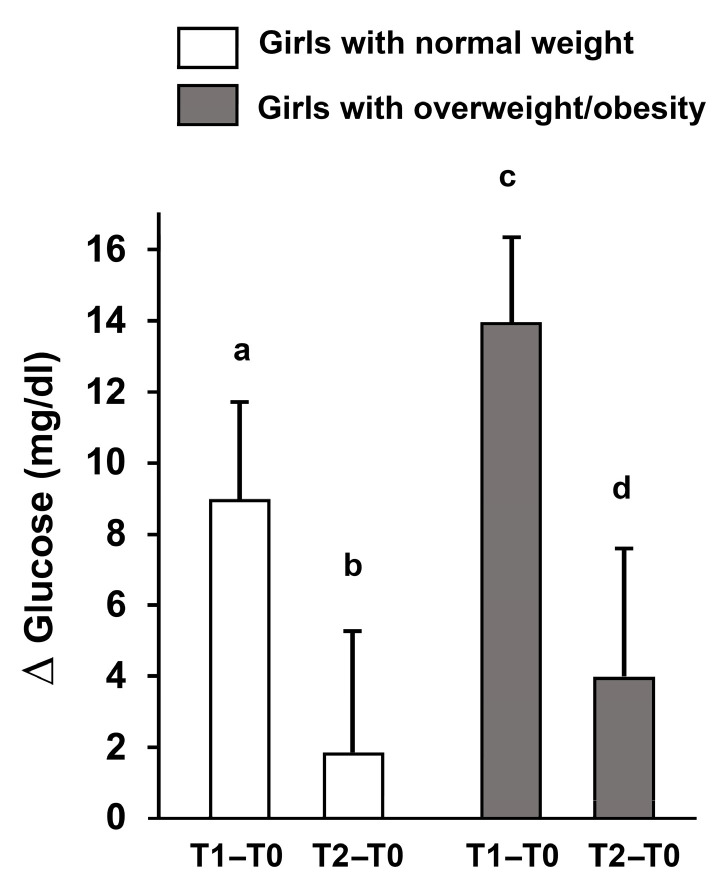
**Changes in fasting glucose (Δ) after acute high-intensity intermittent exercise.** Delta changes in fasting glucose levels immediately post-exercise (T1–T0) and 30 min post-exercise (T2–T0) in adolescent girls with normal weight and overweight/obesity. Data are presented as mean ± S.D. Different letters indicate significant differences between groups or time points according to Bonferroni post hoc tests (*p* < 0.05).

A key finding was that glucose levels in normal-weight girls returned to baseline within 30 min, while they remained elevated in the overweight/obese group, indicating prolonged metabolic stress and possibly altered insulin sensitivity (Figure 3).

**Figure 4:**
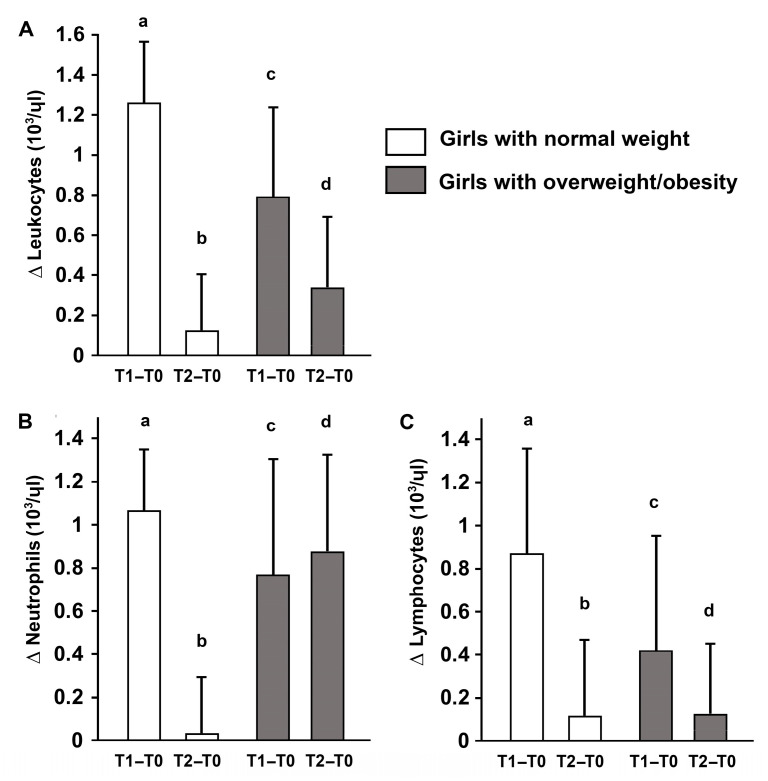
**Changes in total leukocytes and subpopulations (Δ) after acute high-intensity intermittent exercise.** Delta changes in total leukocytes (**A**), neutrophils (**B**), and lymphocytes (**C**) immediately post-exercise (T1–T0) and 30 min post-exercise (T2–T0) in adolescent girls with normal weight and overweight/obesity Data are presented as mean ± S.D. Different letters indicate significant differences between groups or time points according to Bonferroni post hoc tests (*p* < 0.05).

Concurrently, leukocyte subpopulations—including neutrophils and lymphocytes—were analyzed to assess immune responses. Neutrophils increased immediately post-exercise in both groups; however, only participants with overweight/obesity showed persistently elevated neutrophils at 30 min, suggesting a prolonged immune activation. Lymphocyte counts changed transiently after exercise in both groups, reflecting mobilization of the adaptive immune system. These results indicate that body composition influences both the magnitude and temporal dynamics of metabolic and immune responses to acute high-intensity exercise (Figure 4).

## 4. Discussion

We hypothesized that acute exercise would induce transient increases in glucose and leukocyte subpopulations in all participants, while CRP and ESR were included to characterize baseline systemic inflammation rather than acute inflammatory responses. Correlation analyses showed that BMI and body fat were positively associated with glucose, LDL, triglycerides, CRP, ESR, and leukocyte counts, and negatively with HDL, highlighting a link between adiposity and elevated baseline metabolic and inflammatory markers

We further hypothesized that adolescents with overweight/obesity would exhibit greater and more prolonged metabolic and immune responses compared to their normal-weight peers, reflecting heightened baseline metabolic and inflammatory stress.

The present study investigated the acute effects of the Spartacus exercise on metabolic, immune, and inflammatory markers in adolescent girls with normal weight versus overweight/obesity. The main findings can be summarized as follows: (i) fasting glucose and leukocyte counts (total, neutrophils, lymphocytes) increased immediately after exercise in both groups; (ii) in normal-weight participants, these values returned to baseline within 30 min, whereas in adolescents with overweight/obesity, glucose, total leukocytes, and neutrophils remained elevated at T2; (iii) baseline levels of lipids, CRP, ESR, and leukocyte subsets were significantly higher in adolescents with overweight/obesity, reflecting chronic low-grade inflammation, rather than acute exercise-induced inflammatory responses; and (iv) no acute changes were observed in lipid parameters or systemic inflammatory markers.

These findings provide novel insights into the differential acute physiological responses to high-intensity intermittent exercise according to body composition in youth.

Adolescents with overweight/obesity exhibit altered metabolic regulation, including insulin resistance, dyslipidemia, and chronic low-grade systemic inflammation, placing them at elevated cardiometabolic risk from an early age [11,38,39]. Acute high-intensity exercise triggers rapid metabolic adjustments, particularly in glucose regulation and lipid metabolism [20,40], which may differ according to body composition [30,41,42]. Understanding these immediate responses is essential for designing safe and effective exercise programs that maximize benefits while minimizing transient metabolic stress, especially in adolescents with overweight/obesity.

In our study, the Spartacus exercise elicited distinct glucose responses in adolescents with normal weight versus those with overweight/obesity, with fasting glucose increasing immediately after exercise in both groups; however, while normal-weight participants returned to baseline within 30 min, adolescents with overweight/obesity showed a prolonged elevation at T2. These findings of a transient post-exercise increase in glucose, which was particularly prolonged in adolescents with overweight/obesity, are consistent with previous evidence indicating that brief bouts of high-intensity exercise can acutely elevate glycemia, especially in individuals with insulin resistance [40,43]. Adolescents with overweight/obesity therefore experience a more pronounced acute glycemic challenge. This phenomenon parallels the delayed rebound of glycemia observed in healthy adults following short-duration maximal exercise, likely mediated by lactate-driven hepatic gluconeogenesis during recovery [43]. The prolonged glycemic response in adolescents with overweight/obesity may also reflect impaired skeletal muscle glucose uptake and alterations in insulin signaling, including reduced GLUT-4 translocation, as previously described in this population [41,44,45].

Although chronic high-intensity interval training (HIIT) interventions in overweight/obese youth have been shown to improve lipid profile, insulin sensitivity, and adipokine balance [23,46,47,48], acute responses to a single bout of exercise remain variable. Evidence indicates that acute HIIT or maximal exercise can transiently increase glucose and inflammatory markers in adolescents with overweight/obesity, reflecting heightened baseline metabolic and inflammatory stress [19,20]. These findings highlight the importance of considering body composition, recovery timing, and exercise intensity when prescribing high-intensity exercise to youth, to balance acute physiological stress with long-term cardiometabolic benefits [15,18,43].

In contrast to the rapid glycemic responses, acute lipid metabolism appeared largely unaffected by a single bout of Spartacus exercise. No significant changes were observed in total cholesterol, triglycerides, HDL-C, or LDL-C in either group, consistent with previous findings that a single exercise session is generally insufficient to induce measurable lipid changes among sedentary or overweight adolescents [49]. Favorable adaptations in lipid profile and insulin sensitivity are typically achieved through repeated, sustained training interventions over weeks, as demonstrated in adolescents with overweight/obesity following high-intensity interval training programs [23,24,48,50,51]. Together, these results highlight a clear distinction between immediate glycemic responses and longer-term lipid adaptations, emphasizing that acute exercise primarily challenges glucose homeostasis, while lipid improvements require chronic training stimuli.

Adolescent obesity is associated with both metabolic dysregulation and chronic low-grade systemic inflammation, which together contribute to early cardiometabolic risk. Excess adipose tissue acts as an endocrine and immunologically active organ, producing pro-inflammatory cytokines and promoting chronic low-grade systemic inflammation. This inflammatory activation has been widely documented as a key mechanism linking obesity to insulin resistance and glucose dysregulation [11,12,52,53,54].

Therefore, assessing both metabolic and inflammatory responses to acute exercise is essential to better understand the integrated physiological stress induced by high-intensity exercise in adolescents with overweight/obesity.

Acute high-intensity exercise not only challenges metabolic homeostasis but also transiently engages the immune system, potentially influencing systemic inflammation. Adolescents with overweight/obesity are characterized by chronic low-grade systemic inflammation, with elevated baseline levels of inflammatory markers such as CRP and ESR [11,55]. Understanding the acute inflammatory responses to exercise in this population is critical for designing safe and effective interventions.

Despite the acute metabolic stress induced by the Spartacus exercise, CRP and ESR did not change significantly immediately or 30 min following a single session. This finding should be interpreted considering the slow kinetics of these systemic inflammatory markers and the short post-exercise window, rather than as evidence that acute inflammation did not occur. Indeed, CRP and ESR primarily reflect baseline or subacute inflammatory status and are not sensitive to rapid, transient inflammatory signaling induced by a single exercise bout [56].

This interpretation aligns with prior research indicating that systemic inflammatory markers generally respond to long-term, repeated exercise exposure rather than to an isolated acute session. For example, Abassi et al. [22] reported that 12 weeks of HIIT decreased plasma CRP, while ESR remained unchanged in adolescents with obesity. Similarly, a systematic review and meta-analysis by Khalafi et al. [57] demonstrated that reductions in CRP and IL-6 in youth typically occur following sustained exercise programs rather than after a single bout. Baseline inflammatory markers were significantly higher in adolescents with overweight/obesity compared to their normal-weight peers, confirming the presence of chronic low-grade inflammation associated with excess adiposity.

Overall, while high-intensity intermittent exercise acutely challenges metabolic and immune systems, measurable changes in systemic inflammatory markers such as CRP and ESR appear to require repeated or prolonged training stimuli, whereas acute immune responses are more appropriately captured by rapidly responding leukocyte subpopulations [56].

Adolescence is a critical period for metabolic and immune development, with excess adiposity linked to both metabolic disturbances and altered immune function. Adolescents with overweight or obesity often exhibit chronic low-grade systemic inflammation, characterized by elevated circulating pro-inflammatory cytokines (e.g., IL-6, TNF-α) and dysregulated immune cell profiles, including altered leukocyte counts and impaired neutrophil and lymphocyte function [58,59,60,61]. It is plausible that adolescents with overweight/obesity might exhibit an amplified or prolonged immune response to acute high-intensity exercise, due to baseline chronic inflammation and altered leukocyte dynamics. However, empirical evidence in this population remains scarce and inconsistent. Studies such as McMurray et al. [62] show some altered leukocyte and cytokine responses after acute exercise in overweight youth, but results vary depending on marker type, timing, and methodological factors.

In our study, Spartacus exercise induced a significant increase in total leukocytes, neutrophils, and lymphocytes immediately post-exercise (T1) in both adolescents with normal-weight and overweight/obese, consistent with classical exercise-induced leukocytosis [63]. Notably, while normal-weight participants returned to baseline levels by 30 min post-exercise (T2), adolescents with overweight/obesity exhibited sustained elevations in total leukocytes and neutrophils. Lymphocyte counts returned toward baseline in both groups. These findings indicate a prolonged and possibly exaggerated immune activation in adolescents with excess adiposity, likely reflecting heightened baseline systemic inflammation and delayed immune recovery after high-intensity exercise.

Several physiological mechanisms may underlie these observations. Acute exercise stimulates the sympathetic nervous system, promoting catecholamine release that mobilizes leukocytes from the bone marrow and spleen into circulation [56]. Concurrent cortisol secretion further facilitates leukocyte mobilization, while hemodynamic changes and vascular shear stress dislodge immune cells into the bloodstream [64]. Muscle contractions trigger the release of pro-inflammatory cytokines, particularly IL-6, enhancing neutrophil and monocyte mobilization [65]. In adolescents with overweight/obesity, chronic low-grade inflammation and dysregulated immune signaling may delay the normalization of leukocyte counts post-exercise, explaining the prolonged elevations observed [66,67,68].

Taken together, these findings highlight that acute high-intensity exercise triggers complex, intensity-dependent immune responses in adolescents, and that body composition significantly influences both the magnitude and duration of leukocyte changes. This underscores the need for careful monitoring when prescribing high-intensity training to adolescents with overweight/obesity, to balance beneficial immune stimulation with potential excessive inflammatory stress.

This study has several limitations that should be acknowledged. First, the sample size was relatively small and included only female adolescents, which may limit the generalizability of the findings to males or other age groups. Second, the acute design does not allow for conclusions regarding long-term adaptations; chronic training interventions are needed to determine whether repeated Spartacus sessions could elicit sustained metabolic or immune benefits. The short-term nature of the study may have missed delayed peaks or later phases of metabolic and immune responses. Third, the short post-exercise monitoring period (30 min) may have underestimated the magnitude or duration of some physiological responses, particularly for inflammatory markers that often peak several hours after exercise. Fourth, the study assessed only indirect markers of inflammation (CRP, ESR), which were measured primarily at baseline and were not expected to change acutely post-exercise due to their slow kinetics [69,70]. The blood sampling window (immediately post-exercise and 30 min post-exercise) was suitable for capturing acute metabolic and immune cell responses, but not for CRP and ESR, which mainly reflect baseline or subacute inflammatory status rather than acute exercise-induced inflammation. Framing CRP and ESR as acute markers may lead readers to assume that no inflammation occurred, whereas acute inflammatory signaling could not be captured with these biomarkers and the chosen sampling times. A broader panel of biomarkers, including cytokines with faster kinetics (e.g., IL-6, TNF-α, IL-10), is recommended for future studies to provide a more comprehensive understanding of the underlying immunometabolic mechanisms. Fifth, although participants were matched for age and pubertal status, potential variations in diet, sleep, or hormonal fluctuations were not strictly controlled and may have influenced metabolic responses. Additionally, although procedural conditions were standardized, variations in circadian rhythms and procedural stress could have affected outcomes. Measures were taken to minimize these factors (e.g., standardized visit timing and instructions), but residual effects cannot be ruled out.

Finally, individual exercise tolerance was assessed through heart rate monitoring and standardized protocols, but subjective perception of effort (e.g., Borg RPE scale) was not formally recorded. Including such measures could provide valuable insights into inter-individual differences in exercise tolerance and is recommended for future research.

Future research with larger, more diverse cohorts, extended follow-up periods, and a broader panel of biomarkers is warranted to confirm and expand upon these findings.

## 5. Conclusions

In summary, this study demonstrates that acute high-intensity intermittent exercise induces transient increases in glucose and leukocyte counts in adolescent girls, with these responses being more pronounced and prolonged in those with overweight/obesity. These findings indicate that excess adiposity amplifies and delays recovery from the acute metabolic and immune stress induced by vigorous exercise. Overall, the results provide insight into how body composition influences acute physiological responses to high-intensity exercise in youth, highlighting mechanistic differences in metabolic and immune dynamics rather than serving as direct guidance for exercise prescription.

Future research should investigate the chronic adaptations to Spartacus or similar training modalities, focusing on optimizing exercise intensity, duration, and recovery strategies to improve metabolic and immune health in adolescents with overweight or obesity.

## Figures and Tables

**Table 1:** Baseline characteristics of participants.

Variable	Group with Overweight/Obesity (*n* = 16)	Group with Normal Weight (*n* = 14)
**Age (years)**	16.31 ± 1.08	15.86 ± 1.03
**Height (m)**	1.61 ± 0.06	1.62 ± 0.04
**Body mass (kg)**	81.28 ± 12.60 ***	57.76 ± 3.41
**BMI (kg/m^2^)**	31.17 ± 3.85 ***	21.93 ± 0.99
**Body fat (%)**	34.36 ± 3.71 ***	23.77 ± 1.13
**Waist circumference (cm)**	101.88 ± 9.35 ***	69.57 ± 3.90
**Maximal aerobic speed (km/h)**	10.93 ± 0.92 ***	13.44 ± 1.09
**Maximal heart rate (bpm)**	197.29 ± 3.63 **	202.50 ± 4.44

Data are expressed as mean ± SD. ** *p <* 0.01, *** *p <* 0.001; Overweight/obese vs. normal weight.

**Table 2:** Correlation analysis between adiposity indices (BMI and body fat percentage) and metabolic, lipid, and inflammatory markers.

Parameter	BMIr	*p*-Value	Body Fat (%) r	*p*-Value
**Glucose (mg/dL)**	0.660	**<0.001**	0.477	**0.008**
**Total cholesterol (mg/dL)**	0.543	**0.002**	0.541	**0.002**
**Triglycerides (mg/dL)**	0.476	**0.008**	0.439	**0.015**
**HDL-Cholesterol (mg/dL)**	0.557	**0.001**	−0.599	**<0.001**
**LDL-Cholesterol (mg/dL)**	0.600	**<0.001**	0.623	**<0.001**
**Leukocytes (10^3^/µL)**	0.545	**0.002**	0.631	**<0.001**
**Neutrophils (10^3^/µL)**	0.532	**0.002**	0.633	**<0.001**
**Lymphocytes (10^3^/µL)**	0.333	0.072	0.325	0.079
**Monocytes (10^3^/µL)**	0.410	**0.024**	0.475	**0.008**
**Eosinophils (10^3^/µL)**	0.087	0.646	0.056	0.770
**Basophils (10^3^/µL)**	0.018	0.924	0.064	0.737
**C-reactive protein (mg/dL)**	0.628	**<0.001**	0.715	**<0.001**
**Erythrocyte sedimentation rate (mm/h)**	0.705	**<0.001**	0.739	**<0.001**

r = Pearson correlation coefficient. *p*-value indicates statistical significance.

**Table 3:** Acute Metabolic Responses and Baseline Inflammatory Status in Adolescents with Normal Weight versus Overweight/Obesity.

	Normal Weight Group			Overweight/Obese Group			Group Effect			Time Effect			Interaction (TimexGroup		
	T0	T1	T2	T0	T1	T2	F	*p*-Value	η_p_^2^	F	*p*-Value	η_p_^2^	F	*p*-Value	η_p_^2^
**GL (mg/dL)**	82.07 ± 2.37 ***	90.93 ± 3.25 **^×××^**	83.79 ± 4.28	89.44 ± 7.97 ***^#††^	103.44 ± 8.54 **^×××^**^†††^	93.50 ± 8.03 ^†††^	22.852	**<0.001**	0.449	74.385	**<0.001**	0.727	3.491	**0.037**	0.111
**TC (mg/dL)**	161.73 ± 10.36	164.47 ± 10.21 **^×^**	162.16 ± 8.03	175.44 ± 15.36 ^††^	178.65 ± 13.57 ^††^	177.74 ± 14.02 ^††^	11.722	**0.002**	0.295	2.308	0.109	0.076	0.244	0.784	0.009
**TG (mg/dL)**	89.92 ± 15.91 **	92.89 ± 15.02	90.70 ± 15.19	104.64 ± 13.89 ^†^	108.77 ± 17.75 ^†^	107.17 ± 14.69 ^††^	8.970	**0.006**	0.243	1.838	0.169	0.062	0.114	0.892	0.004
**HDLC (mg/dL)**	54.47 ± 6.44	55.87 ± 6.03	54.92 ± 5.99	43.48 ± 3.82 ^†††^	44.73 ± 5.20 ^†††^	44.01 ± 4.97 ^†††^	35.370	**<0.001**	0.558	2.320	0.108	0.077	0.016	0.984	0.001
**LDLC (mg/dL)**	89.28 ± 13.63	90.02 ± 11.17	89.10 ± 10.97	111.03 ± 14.70 ^†††^	112.17 ± 14.20 ^†††^	112.30 ± 15.32 ^†††^	22.511	**<0.001**	0.446	0.256	0.775	0.009	0.161	0.851	0.006
**LE (10^3^/µL)**	5.44 ± 0.88 ***	6.69 ± 0.60 ^×××^	5.56 ± 0.93	7.87 ± 1.86 ***^#†††^	8.66 ± 1.94 ^×††^	8.21 ± 1.69 ^†††^	21.194	**<0.001**	0.431	34.782	**<0.001**	0.554	3.763	**0.029**	0.118
**NE (10^3^/µL)**	2.87 ± 0.56 ***	3.93 ± 0.51 ^×××^	2.91 ± 0.53	4.45 ± 1.53 ^#††^	5.22 ± 2.31 ^†^	5.33 ± 1.99 ^†††^	13.672	**<0.001**	0.328	8.157	**<0.001**	0.226	3.299	**0.044**	0.105
**LYM (10^3^/µL)**	2.01 ± 0.44 ***	2.88 ± 0.52 ^×××^	2.13 ± 0.35	2.63 ± 0.62 **^††^	3.05 ± 0.56	2.76 ± 0.46 ^†††^	11.036	**0.002**	0.283	23.301	**<0.001**	0.454	3.447	**0.039**	0.110
**MONO (10^3^/µL)**	0.39 ± 0.12 *	0.45 ± 0.10	0.43 ± 0.09	0.56 ± 0.18 ^††^	0.60 ± 0.14 ^††^	0.58 ± 0.12 ^†††^	14.646	**<0.001**	0.343	2.210	0.119	0.073	0.137	0.872	0.005
**EO (10^3^/µL)**	0.18 ± 0.10	0.16 ± 0.07	0.17 ± 0.09	0.21 ± 0.15	0.18 ± 0.14	0.20 ± 0.14	0.366	0.550	0.013	2.562	0.086	0.084	0.085	0.919	0.003
**BASO (10^3^/µL)**	0.033 ± 0.01	0.030 ± 0.02	0.030 ± 0.01	0.040 ± 0.16	0.037 ± 0.01	0.038 ± 0.01	4.602	**0.041**	0.141	0.600	0.552	0.021	0.003	0.997	0.000
**CRP (mg/dL)**	0.35 ± 0.20	0.39 ± 0.23	0.42 ± 0.23	2.50 ± 1.14 ^†††^	2.55 ± 1.10 ^†††^	2.68 ± 1.20 ^†††^	57.207	**<0.001**	0.671	0.987	0.379	0.034	0.300	0.742	0.011
**ESR (mm/h)**	3.93 ± 1.86	4.07 ± 1.38	4.37 ± 1.40	13.38 ± 3.50 ^†††^	13.44 ± 3.56 ^†††^	13.69 ± 2.87 ^†††^	108.80	**<0.001**	0.795	0.747	0.479	0.026	0.022	0.979	0.001

Values are presented as mean ± SD. T0: pre-test; T1: immediately post-test; T2: 30 min post-test. GL: glucose; TC: total cholesterol; TG: triglycerides; HDL-C: high-density lipoprotein cholesterol; LDL-C: low-density lipoprotein cholesterol; CRP: C-reactive protein; ESR: erythrocyte sedimentation rate; LE: leukocytes; NE: neutrophils; LYM: lymphocytes; MONO: monocytes; EO: eosinophils; BASO: basophils. Group effect, time effect, and time × group interaction was analyzed using two-way repeated-measures ANOVA. * *p* < 0.05, ** *p* < 0.01, *** *p* < 0.001 T0 vs. T1; ^#^ *p* < 0.05 T0 vs. T2; ^×^ *p* < 0.05, ^×××^ *p* < 0.001 T1 vs. T2 using paired-sample *t*-tests. ^†^ *p* < 0.05, ^††^ *p* < 0.01, ^†††^ *p* < 0.001 vs. the normal-weight group at the same time point using independent-sample *t*-tests. Note: CRP and ESR values represent baseline inflammatory status. Due to their slow kinetics, these markers do not reflect acute exercise-induced inflammatory responses, unlike glucose and leukocyte counts, which respond rapidly to short-term exercise (Gray background).

## Data Availability

The data that support the findings of this study are available from the corresponding author upon reasonable request.

## Abbreviations

The following abbreviations are used in this manuscript:

GL	Glucose
TC	Total cholesterol
TG	Triglycerides
HDL-C	High-density lipoprotein cholesterol
LDL-C	Low-density lipoprotein cholesterol
CRP	C-reactive protein
ESR	Erythrocyte sedimentation rate
LE	Leukocytes
NE	Neutrophils
LYM	Lymphocytes
MONO	Monocytes
EO	Eosinophils
BASO	Basophils
HIIT	High-intensity interval training
